# Modeling the Nonlinear Deformation of Highly Porous Cellular Plastics Filled with Clay Nanoplatelets

**DOI:** 10.3390/ma15031033

**Published:** 2022-01-28

**Authors:** Aivars Lagzdiņš, Alberts Zilaucs, Ilze Beverte, Jānis Andersons

**Affiliations:** Institute for Mechanics of Materials, University of Latvia, 3 Jelgavas St., LV-1004 Riga, Latvia; aivars.lagzdins@lu.lv (A.L.); alberts.zilaucs@apollo.lv (A.Z.); ilze.beverte@lu.lv (I.B.)

**Keywords:** cellular plastic, porosity, polyurethane foams, nanoplatelets, structural element, anisotropy, orientational averaging

## Abstract

Rigid low-density plastic foams subjected to mechanical loads typically exhibit a nonlinear deformation stage preceding failure. At moderate strains, when the geometrical nonlinearity is negligible, such foam response is predominantly caused by the nonlinearity of deformation of their principal structural elements—foam struts. Orientational averaging of stresses in foam struts enables estimation of the stresses taken up by foams at a given applied strain. Based on a structural model of highly porous anisotropic cellular plastics filled with clay nanoplatelets and the orientational averaging, a method for calculating their nonlinear deformation is derived in terms of structural parameters of the porous material, the mechanical properties of the monolithic polymer, and filler particles and their spatial orientation. The method is applied to predicting the tensile stress-strain diagrams of organoclay-filled low-density rigid polyurethane foams, and reasonable agreement with experimental data is demonstrated.

## 1. Introduction

The aim of this research was to develop a structural model for calculating the nonlinear deformation of highly porous cellular plastics with a porosity Π (0 < Π < 1) exceeding 90%. Such cellular plastics (i.e., plastic foams) are used in various fields of engineering, e.g., as damping and heat- and sound-insulating materials. They are not meant to carry high mechanical loads, but their stiffness and strength have to be sufficiently high for intended applications. Filling the plastic foams with micro- or nanoparticles enables improving their functional properties without interfering adversely with foam morphology.

When anisometric filler particles are used in production of low-density composite foams, they tend to align with the principal flow direction of the liquid chemical formulation during foaming as well as with the stretch directions of cell walls and struts, as demonstrated for polymer foams with rod- [1,2,3,4,5,6] and plate-like [7,8,9] mico- and nanofillers. Interactions between dispersed carbon fibers and growing cells in high-pressure foam injection molding experiments using a polystyrene/carbon fiber/carbon dioxide system was investigated in [3], by an in situ visualization technique. It was found that the fibers in close proximity to the growing cells exhibited both translational and rotational displacements. An analytical model was developed in [4] to describe the instantaneous location and angle of rod-like conductive fillers as affected by cell growth during the foaming of conductive polypropylene composites. The reorientation of nanoclay particles during the foaming process has been considered, and its effect on the stiffness of the cell walls was modeled in [9].

The nonlinear mechanical response of such low-density polymer foams can be described by either applying a constitutive material model needing calibration based on foam tests (see, e.g., in [10]), or by a structural model relating foam properties to their morphology and mechanical properties of the respective monolithic polymer. In low-density closed-cell polyurethane (PUR) foams, most of the polymer (more than 90%, see, e.g., in [11]) is contained within cell struts. Such morphology enables accurate prediction of the elastic properties of anisotropic foams by considering the deformation of a regular elementary cell composed of struts, such as parallelepiped [12,13], Kelvin [14,15], and generalized Kelvin [16,17,18] cells. Alternatively, models based on orientation distribution of foam struts are used [19,20]. Concerning the nonlinear deformation, the behavior of a large network of cells under compression has been simulated numerically by a finite element analysis employing either regular [21] or disordered cell geometry. In the latter case, both random generated cellular microstructure [22] and the actual morphology obtained by 3D scanning of foams [23] have been considered. The application of strut-based models also has been mostly confined to the compressive response, when the nonlinearity is caused by a progressive buckling of the struts [19,24]. In this study, we consider the effect of nonlinear deformation of foam strut material on the foam response.

The aim of the present study is to create a mathematical model for describing the nonlinear deformation of highly porous cellular plastics filled with clay nanoplatelets, taking into account the influence of the spatial alignment of filler particles in foam struts. To this end, we utilize the method developed in [20,25,26] for predicting the elastic properties of foams by means of orientational averaging of the linear elastic characteristics of their structural elements, and extend it to nonlinear deformation of structural elements. The paper is organized as follows. Load-carrying and structural elements of low-density foams, calculation of stiffness tensor of the composite foam strut material, and evaluation of the nonlinear deformation of foams by orientation averaging of the stress–strain response of the structural elements are presented in Section 2. Analytical approximations of the nonlinear axial stress–strain relation of the load-carrying element of foams are described in Section 3, and application of the model to prediction of the mechanical response in uniaxial tension of rigid low-density PUR foams with different loadings of nanoclay filler is demonstrated in Section 4.

## 2. Structural Model

**Figure 1:**
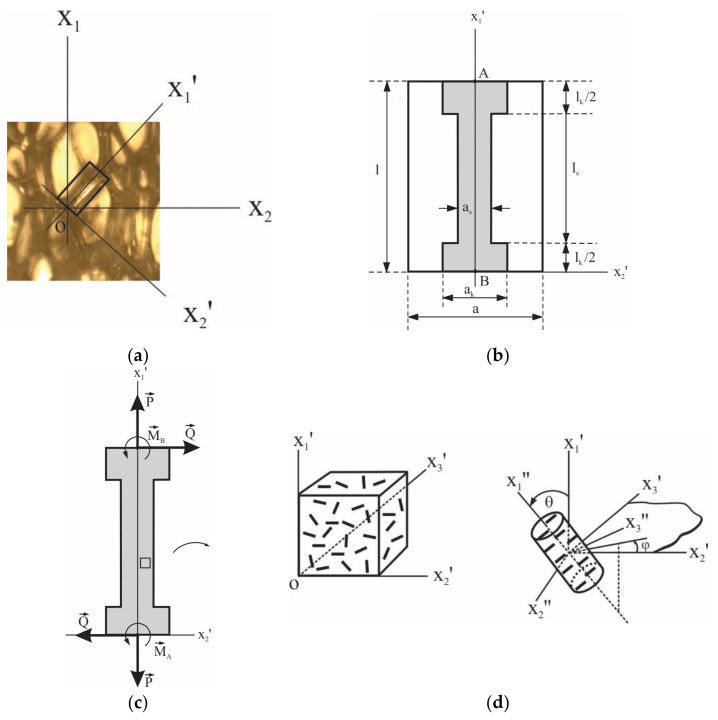
Highly porous cellular plastic: (**a**) Micrography showing the reticulated morphology of foams and global (i.e., foam) and local (strut) coordinate systems; (**b**) Schematic of the structural element comprising the load-carrying element (gray); (**c**) Schematic of the load-carrying element showing the loads applied to it; (**d**) Calculation element for estimation of stiffness tensor C of the nanoplatelet-filled material of load-carrying element.

We will consider a cellular plastic of porosity Π ≥ 90% filled with clay particles in the form of nanoplatelets. Owing to the high porosity, we assume that all mass of the polymer is concentrated in rod-like struts and knots of the foams, as seen in the micrograph Figure 1a, and cell walls are too thin to take up a significant mechanical load. The effect of gas pressure in the closed cells on foam deformation can be neglected at small strains [20]. Then the external loads are taken up only by the struts and knots.

The load-carrying element of such a cellular plastic, as in [20,25], is taken in the form of a straight shaft with thickened end parts, depicted in Figure 1b,c. The cross section of the shaft is an equilateral triangle of side $a_{s}$. The thickened parts are rectangular parallelepipeds with a side $a_{k}$ and height $l_{k}/2$. The load-carrying elements are connected to each other by their ends in knots and form a spatial structure able to resist various mechanical actions. This element is placed in a coaxial rectangular parallelepiped of length $l=l_{s}+l_{k}$ and a square cross section with a side $a={\left[\frac{V_{l}}{\left(1-\Pi \right)l}\right]}^{\frac{1}{2}}$, where $V_{l}$ is volume of the load-carrying element. In such a way, we arrive at the structural element of the foam, Figure 1b.

The load-carrying element takes up axial forces *P*, transverse forces *Q*, and moments $M_{A}=M_{B}=Ql/2$ (see Figure 1c), and, as a result, it changes its length and transverse dimensions, undergoes shear deformations, and bends. The transverse deformation of the load-carrying element practically does not affect that of the structural element, but bending loads add to it a supplementary shear deformation. Considering that the second moments of cross-sectional area of the triangular shaft about the $x_{2}^{\prime }$ and $x_{3}^{\prime }$ axes coincide, the stiffness tensor ${\overset{*}{C}}^{lce}$ of the load-carrying element has only three nonzero components in the $x_{i}^{\prime }$ axes—${\overset{*}{C}}_{1111}^{lce}$ and ${\overset{*}{C}}_{1212}^{lce}={\overset{*}{C}}_{1313}^{lce}$. Their magnitude depends on the geometrical dimensions of the load-carrying element and the elastic characteristics of its material [20,25,26].

### 2.1. Stiffness of the Composite Material of the Load-Carrying Element

The polymer material of load-carrying elements is uniformly filled with clay nanoplatelets around the longitudinal axis $x_{1}^{\prime }$ with a volume fraction $V_{f}$. As a result, the element is transversely isotropic. The stiffness tensor C of its material is found by spatially averaging the transversely isotropic stiffness tensor $\overset{*}{C}$ of the calculation element (see, e.g., in [27])

(1) $$C=\int_{O}f\left(g\right)\overset{*}{C}\left(g\right)dg,\;g\in O,$$

where *O* is the group of 3D orientations of the elements. Their orientation distribution function $f\left(g\right)$ is taken in the form:

(2) $$f\left(g\right)=f\left(\xi \right)=\frac{2n+1}{\lambda_{1}+{2\lambda }_{23}}\left[\lambda_{1}\xi_{1}^{2n}+2\frac{\left(2n-1\right)!!}{\left(2n\right)!!}\lambda_{23}{\left(1-\xi_{1}^{2}\right)}^{n}\right],\;n\geq 0$$

where $\lambda_{1}$ and $\lambda_{23}$ are nonnegative parameters such that $\lambda_{1}+\lambda_{23}=1$. The unit vector ξ indicates the spatial directions of calculation elements, with $\xi_{1}=\cos\theta$ , where θ is the angle between it and the axis $x_{1}^{\prime }$ of the load-carrying element, see Figure 1d.

For example, if $\lambda_{23}$ = 0, then $\lambda_{1}=1$ and the distribution function Equation (2) has only one parameter, n; performing integration (1), we have

(3) $$\begin{array}{c} \begin{array}{c} C_{1111}=k\left[\left(2n+3\right)\left(2n+1\right){\overset{*}{C}}_{1111}+3\left({\overset{*}{C}}_{2222}+{\overset{*}{C}}_{3333}\right)+2{\overset{*}{C}}_{2233}+4{\overset{*}{C}}_{2323}+\right.\\ \left.2\left(2n+1\right)\left({\overset{*}{C}}_{1122}+{\overset{*}{C}}_{3311}+2{\overset{*}{C}}_{1212}+2{\overset{*}{C}}_{3131}\right)\right], \end{array}\\ \begin{array}{c} C_{2222}=C_{3333}=k\left[3{\overset{*}{C}}_{1111}+\frac{1}{2}\left(n+2\right)\left(n+1\right)(3{\overset{*}{C}}_{2222}+3{\overset{*}{C}}_{3333}+2{\overset{*}{C}}_{2233}+\right.\\ \left.4{\overset{*}{C}}_{2323})+2\left(n+1\right)\left({\overset{*}{C}}_{1122}+{\overset{*}{C}}_{3311}+2{\overset{*}{C}}_{1212}+2{\overset{*}{C}}_{3131}\right)\right], \end{array}\\ \begin{array}{c} C_{1122}=C_{3311}=k\left[\left(2n+1\right)({\overset{*}{C}}_{1111}-2{\overset{*}{C}}_{1212}+{\overset{*}{C}}_{3131})+\left(n+1\right)({\overset{*}{C}}_{2222}+\right.\\ \left.{\overset{*}{C}}_{3333})+2\left(n+2\right){\overset{*}{C}}_{2233}-2{\overset{*}{C}}_{2323}+\left(2n^{2}+5n+4\right)\left({\overset{*}{C}}_{1122}+{\overset{*}{C}}_{3311}\right)\right], \end{array}\\ \begin{array}{c} C_{2233}=k\left[{\overset{*}{C}}_{1111}-2{\overset{*}{C}}_{1212}-2{\overset{*}{C}}_{3131}+\frac{1}{2}\left(n+2\right)\left(n+1\right)\left({\overset{*}{C}}_{2222}+{\overset{*}{C}}_{3333}\right)+\right.\\ \left(3n^{2}+9n+4\right){\overset{*}{C}}_{2233}-2\left(n+3n+1\right){\overset{*}{C}}_{2323}+2\left(n+2\right)\left.\left({\overset{*}{C}}_{1122}+{\overset{*}{C}}_{3311}\right)\right], \end{array}\\ \begin{array}{c} C_{1212}=C_{3131}=k\left[\left(2n+1\right)\left({\overset{*}{C}}_{1111}-{\overset{*}{C}}_{1122}-{\overset{*}{C}}_{3311}\right)+\left(n+1\right)({\overset{*}{C}}_{2222}+\right.\\ {\overset{*}{C}}_{3333})-{\overset{*}{C}}_{2233}+\left(2n+3\right){\overset{*}{C}}_{2323}+\left(2n^{2}+3n+3\right)\left.\left({\overset{*}{C}}_{1212}+{\overset{*}{C}}_{3131}\right)\right] \end{array}\\ \begin{array}{c} C_{2323}=k\left[{\overset{*}{C}}_{1111}-{\overset{*}{C}}_{1122}-{\overset{*}{C}}_{3311}+\frac{1}{2}\left(n+2\right)\left(n+1\right)\left({\overset{*}{C}}_{2222}+{\overset{*}{C}}_{3333}\right)\right.+\\ \left(n^{2}+3n+1\right){\overset{*}{C}}_{2233}+\left(2n+6n+3\right){\overset{*}{C}}_{2323}+\left(2n+3\right)\left.\left({\overset{*}{C}}_{1212}+{\overset{*}{C}}_{3131}\right)\right], \end{array}\\ k=\frac{1}{\left(2n+5\right)\left(2n+3\right)}. \end{array}$$

Using the Halpin–Pagano–Tsai equations modified for the case of platelike filler particles [28], the stiffness ${\overset{*}{C}}_{ijkl}$ is expressed in the form

(4) $$\begin{array}{c} {\overset{*}{C}}_{1111}=\frac{1-{\overset{*}{\nu }}_{23}^{2}}{{\overset{*}{E}}_{2}^{2}{\overset{*}{\Delta }}_{1}},\;{\overset{*}{C}}_{2222}={\overset{*}{C}}_{3333}=\frac{1-{\overset{*}{\nu }}_{12}{\overset{*}{\nu }}_{21}}{{\overset{*}{E}}_{1}{\overset{*}{E}}_{2}{\overset{*}{\Delta }}_{1}},\\ {\overset{*}{C}}_{1122}={\overset{*}{C}}_{3311}=\frac{{\overset{*}{\nu }}_{21}(1+{\overset{*}{\nu }}_{23})}{{\overset{*}{E}}_{1}{\overset{*}{E}}_{2}{\overset{*}{\Delta }}_{1}}=\frac{{\overset{*}{\nu }}_{12}(1+{\overset{*}{\nu }}_{23})}{{\overset{*}{E}}_{2}^{2}{\overset{*}{\Delta }}_{1}},\;{\overset{*}{C}}_{2233}=\frac{{\overset{*}{\nu }}_{23}+{\overset{*}{\nu }}_{12}{\overset{*}{\nu }}_{21}}{{\overset{*}{E}}_{1}{\overset{*}{E}}_{2}{\overset{*}{\Delta }}_{1}},\\ {\overset{*}{C}}_{1212}={\overset{*}{C}}_{3131}={\overset{*}{G}}_{12}={\overset{*}{G}}_{31},\;{\overset{*}{C}}_{2323}={\overset{*}{G}}_{23}=\frac{{\overset{*}{E}}_{2}}{2(1+{\overset{*}{\nu }}_{23})},\\ {\overset{*}{\Delta }}_{1}=\frac{1}{{\overset{*}{E}}_{1}{\overset{*}{E}}_{2}^{2}}\left(1+{\overset{*}{\nu }}_{23}\right)\left(1-{\overset{*}{\nu }}_{23}-2{\overset{*}{\nu }}_{12}{\overset{*}{\nu }}_{21}\right), \end{array}$$

with

(5) $$\begin{array}{c} {\overset{*}{E}}_{1}=\frac{1+\xi^{\prime }\eta^{\prime }V_{f}}{1-\eta^{\prime }V_{f}}E_{m},\;{\overset{*}{E}}_{2}={\overset{*}{E}}_{3}=\frac{1+\xi^{\prime \prime }\eta^{\prime \prime }V_{f}}{1-\eta^{\prime \prime }V_{f}}E_{m},\\ {\overset{*}{G}}_{12}={\overset{*}{G}}_{31}=\frac{1+\chi^{\prime }\lambda^{\prime }V_{f}}{1-\lambda^{\prime }V_{f}}G_{m},\;{\overset{*}{G}}_{23}=\frac{1+\chi^{\prime \prime }\lambda^{\prime \prime }V_{f}}{1-\lambda^{\prime \prime }V_{f}}G_{m},\\ {\overset{*}{\nu }}_{12}={\overset{*}{\nu }}_{13}=\nu_{f}V_{f}+\nu_{m}\left(1-V_{f}\right). \end{array}$$

Here,

(6) $$\begin{array}{c} \eta^{\prime }=\frac{E_{f}/E_{m}-1}{E_{f}/E_{m}+\xi^{\prime }},\;\eta^{\prime \prime }=\frac{E_{f}/E_{m}-1}{E_{f}/E_{m}+\xi^{\prime \prime }},\\ \lambda^{\prime \prime }=\frac{G_{f}/G_{m}-1}{G_{f}/G_{m}+\chi^{\prime \prime }},\;\lambda^{\prime \prime }=\frac{G_{f}/G_{m}-1}{G_{f}/G_{m}+\chi^{\prime \prime }},\\ \xi^{\prime }=2,\;\chi^{\prime }=1,\;\xi^{\prime \prime }=\chi^{\prime \prime }=\frac{2D}{h} \end{array}$$

In the above relations, $V_{f}$ is the volume fraction of filler (clay nanoplatelets), $\nu_{f}$ and $\nu_{m}$ are the Poisson ratios, $E_{f}$ and $E_{m}$ are the elastic moduli, and $G_{f}$ and $G_{m}$ are the shear moduli of the filler and polymer matrix, respectively. *D* and *h* are the diameter and thickness, respectively, of clay nanoplatelets.

### 2.2. Stresses in the Structural Element

The stresses $\overset{*}{\sigma_{11}}$, $\overset{*}{\sigma_{12}}$, and $\overset{*}{\sigma_{13}}$ in the structural element, considering the porosity Π of the foam, are found as follows:

(7) $$\begin{array}{c} \overset{*}{\sigma_{11}}={\left(1-\Pi \right)}^{\alpha }\frac{1-{\overset{*}{\nu }}_{12}}{\left(1+{\overset{*}{\nu }}_{12}\right)\left(1-2{\overset{*}{\nu }}_{13}\right)}{\left(\frac{l_{k}}{l}\frac{F_{l}}{F_{k}}+\frac{l_{s}}{l}\frac{F_{l}}{F_{s}}\right)}^{-1}\overset{*}{\sigma },\\ \overset{*}{\sigma_{12}}=\left(1-\Pi \right){\overset{*}{G}}_{0}\overset{*}{\varepsilon_{12}},\;\overset{*}{\sigma_{13}}=\left(1-\Pi \right){\overset{*}{G}}_{0}\overset{*}{\varepsilon_{13}}, \end{array}$$

where $\overset{*}{\sigma }=f\left({\overset{*}{\varepsilon }}_{11}\right)$ is the stress of the material of load-carrying element (i.e., clay nanoplatelet-filled polymer) as a function of strain in loading along the axis $x_{1}^{\prime }$, and [20,25]

(8) $${\overset{*}{G}}_{0}=\frac{2}{{\overset{*}{E}}_{1}}\cdot \frac{l_{s}}{l}{\left(\frac{l_{s}}{a_{s}}\right)}^{2}\frac{F_{l}}{F_{s}}+\frac{\left[1+{\left(\frac{l_{s}}{l}\right)}^{3}\right]\frac{F_{l}}{F_{s}}}{{\left(\frac{a_{k}}{l_{k}}\cdot \frac{l_{k}}{l}\right)}^{2}{\overset{*}{E}}_{1}}+\frac{\chi }{{\overset{*}{E}}_{1}}+\frac{1}{{\overset{*}{G}}_{12}}{\left(\frac{l_{k}}{l}\cdot \frac{F_{l}}{F_{k}}+\frac{l_{s}}{l_{k}}\cdot \frac{F_{l}}{F_{s}}\right)}^{-1}.$$

In Equation (8), ${\overset{*}{E}}_{1}$ and ${\overset{*}{G}}_{12}$ are the elastic and shear moduli of the platelet-filled material of the load-carrying element (determined from stiffness tensor C of the composite given by Equation (1)) and

(9) $$\begin{array}{c} \frac{F_{l}}{F_{k}}=\left[\frac{\sqrt{3}}{4}{\left(\frac{a_{s}}{l_{s}}\right)}^{2}{\left(\frac{l_{s}}{l}\right)}^{3}+{\left(\frac{a_{k}}{l_{k}}\right)}^{2}{\left(\frac{l_{k}}{l}\right)}^{3}\right]{\left(\frac{a_{k}}{l_{k}}\cdot \frac{l_{k}}{l}\right)}^{-2},\\ \frac{F_{l}}{F_{s}}=\left[{\left(\frac{a_{s}}{l_{s}}\right)}^{2}{\left(\frac{l_{s}}{l}\right)}^{3}+\frac{4}{\sqrt{3}}{\left(\frac{a_{s}}{l_{k}}\right)}^{2}{\left(\frac{l_{k}}{l}\right)}^{3}\right]{\left(\frac{a_{s}}{l_{s}}\cdot \frac{l_{s}}{l}\right)}^{-2},\\ \frac{a_{k}}{a_{s}}=\frac{\frac{a_{k}}{l_{s}}}{\frac{l_{s}}{l}\cdot \frac{a_{s}}{l_{s}}},\;\frac{l_{k}}{l_{s}}=\frac{\frac{l_{k}}{l}}{1-\frac{l_{k}}{l}},\;\frac{l_{s}}{l}=1-\frac{l_{k}}{l},\\ \chi =\frac{1}{2}\chi_{0}{\overset{*}{E}}_{1}V_{l},\;\chi_{0}\geq 0. \end{array}$$

It is seen that the stresses $\overset{*}{\sigma_{11}}$, $\overset{*}{\sigma_{12}}$, and $\overset{*}{\sigma_{13}}$ depend on the non-dimensional parameters $\frac{a_{s}}{l_{s}}$, $\frac{a_{k}}{l_{k}}$, $\frac{l_{k}}{l}$, χ, and $\Pi =1-\frac{\rho }{\rho_{m}}$, where ρ and $\rho_{m}$ are densities of the porous material and matrix, respectively, the power α is introduced to consider the fact that, under linear deformations, the open-cell foam can change its volume and, thus, the density ρ.

Taking into account that approximately $\frac{a_{k}}{a_{s}}\approx$ 1.5 and $a_{k}\approx l_{k}$, we obtain that

$$\frac{l_{k}}{l}\approx \frac{\frac{a_{s}}{l_{s}}}{\frac{a_{s}}{l_{s}}+0.77},\;\frac{l_{s}}{l}\approx \frac{0.77}{\frac{a_{s}}{l_{s}}+0.77}.$$

The parameter $\frac{a_{s}}{l_{s}}$ is found by numerically solving the nonlinear equation [20,25]

$$1-\Pi =\frac{\rho }{\rho_{m}}=\frac{{\left(\frac{a_{s}}{l_{s}}\right)}^{2}}{{\left(\frac{a_{s}}{l_{s}}+0.77\right)}^{3}}\left(\frac{3\sqrt{3}}{4}0.77^{3}+\frac{a_{s}}{l_{s}}\right).$$

### 2.3. Evaluation of Foam Stresses and Foam Stiffness

Now that the composition of the structural element of foam is known, the stresses $\sigma_{ij}$ of the deformed porous material are calculated by orientationally averaging the stresses $\overset{*}{\sigma_{11}}$, $\overset{*}{\sigma_{12}}$, and $\overset{*}{\sigma_{13}}$, Equation (7), of the transversely isotropic structural elements with account of their orientational distribution function $f_{p}\left(\xi \right)$.

Assuming that [20]

(10) $$f_{p}\left(\xi \right)=\frac{1}{k}\left[1+\left(k^{3}-1\right)\xi_{1}^{2n}\right],\;\xi_{1}=\cos\theta ,\;n=\frac{k\left(k+1\right)}{2},\;k>0,$$

where k is the extension degree of pores, we write that

(11) $$\begin{array}{ll} \sigma_{ij}= & \int_{O}f_{p}\left(\xi \right)\left[l_{1i}l_{ij}\overset{*}{\sigma_{11}}+\left(l_{1i}l_{2j}+l_{1j}l_{2i}\right)\overset{*}{\sigma_{12}}\right]dg\\ & \;\;\;\;=\frac{1}{S}\int f_{p}\left(\xi \right)\left\{l_{1i}l_{ij}\overset{*}{\sigma_{11}}\right.\\ & \;\;\;\;+\left.\frac{1}{2}\left[\left(l_{1i}l_{2j}+l_{1j}l_{2i}\right)\overset{*}{\sigma_{12}}+\left(l_{1i}l_{3j}+l_{1j}l_{3i}\right)\overset{*}{\sigma_{13}}\right]\right\}ds, \end{array}$$

where $\overset{*}{\sigma_{11}}$ is a nonlinear function, calculated by Equation (7) using $\overset{*}{\sigma }=f\left({\overset{*}{\varepsilon }}_{11}\right)$ found from experiments,

$$\overset{*}{\sigma_{12}}=2{\overset{*}{C}}_{1212}^{p}{\overset{*}{\varepsilon }}_{12},\;\overset{*}{\sigma_{13}}=2{\overset{*}{C}}_{1313}^{p}{\overset{*}{\varepsilon }}_{13},\;{\overset{*}{C}}_{1212}^{p}={\overset{*}{C}}_{1313}^{p},\;l_{ij}=\cos\left(x_{i}^{\prime },x_{j}\right),\;ds=\frac{1}{4\pi }.$$

The evaluation of integral (11) can be simplified assuming that the Euler angles $\varphi_{2}=0$ and $\varphi_{1}=\frac{\pi }{2}-\varphi$, where φ is the spherical angle. Then,

(12) $$\begin{array}{c} l_{11}=\cos\theta ,\;l_{12}=\cos\varphi \sin\theta ,\;l_{13}=\sin\varphi \sin\theta ,\;l_{21}=0,\;l_{22}=\sin\varphi ,\\ l_{23}=-\cos\varphi ,\;l_{31}=-\sin\theta ,\;l_{32}=\cos\varphi \cos\theta ,\;l_{33}=\sin\varphi \cos\theta ,\\ 0\leq \varphi <2\pi ,\;0\leq \theta <\pi , \end{array}$$

and in the linear case, we have the following expressions for components of the stiffness tensor $C_{p}$ of the porous material [20]

(13) $$\begin{array}{c} C_{1111}^{p}=\frac{1}{k}\left\{\left[\frac{1}{5}+\frac{\left(2n+1\right)\left(k-1\right)}{2n+5}\right]\right.{\overset{*}{C}}_{1111}^{p}+\left.8\left[\frac{1}{15}+\frac{\left(2n+1\right)\left(k-1\right)}{\left(2n+5\right)\left(2n+3\right)}\right]{\overset{*}{C}}_{1212}^{p}\right\},\\ C_{2222}^{p}=C_{3333}^{p}=\frac{1}{k}\left\{\left[\frac{1}{5}+\frac{3\left(k-1\right)}{\left(2n+5\right)\left(2n+3\right)}\right]\right.{\overset{*}{C}}_{1111}^{p}+\left.8\left[\frac{1}{15}+\frac{\left(2n+1\right)\left(k-1\right)}{\left(2n+5\right)\left(2n+3\right)}\right]{\overset{*}{C}}_{1212}^{p}\right\},\\ C_{1122}^{p}=C_{3311}^{p}=\frac{1}{k}\left\{\left[\frac{1}{15}+\frac{\left(2n+1\right)\left(k-1\right)}{\left(2n+5\right)\left(2n+3\right)}\right]\left({\overset{*}{C}}_{1111}^{p}-4{\overset{*}{C}}_{1212}^{p}\right)\right\},\\ C_{2233}^{p}=\frac{1}{k}\left\{\left[\frac{1}{15}+\frac{k-1}{\left(2n+5\right)\left(2n+3\right)}\right]\left({\overset{*}{C}}_{1111}^{p}-4{\overset{*}{C}}_{1212}^{p}\right)\right\},\\ C_{1212}^{p}=C_{3131}^{p}=\frac{1}{k}\left\{\left[\frac{1}{15}+\frac{\left(2n+1\right)\left(k-1\right)}{\left(2n+5\right)\left(2n+3\right)}\right]\right.{\overset{*}{C}}_{1111}^{p}+\left.2\left[\frac{1}{5}+\frac{\left(2n^{2}+3n+3\right)\left(k-1\right)}{\left(2n+5\right)\left(2n+3\right)}\right]{\overset{*}{C}}_{1212}^{p}\right\},\\ C_{2323}^{p}=\frac{1}{k}\left\{\left[\frac{1}{15}+\frac{k-1}{\left(2n+5\right)\left(2n+3\right)}\right]\right.{\overset{*}{C}}_{1111}^{p}+\left.2\left(\frac{1}{5}+\frac{k-1}{2n+5}\right){\overset{*}{C}}_{1212}^{p}\right\}, \end{array}$$

$C_{2222}^{p}-C_{2233}^{p}=2C_{2323}^{p}$, where ${\overset{*}{C}}_{1111}^{p}$ and ${\overset{*}{C}}_{1212}^{p}$ are the stiffnesses of structural elements,

(14) $$\begin{array}{c} E_{1}^{p}=\frac{\Delta }{{\left(C_{2222}^{p}\right)}^{2}-{\left(C_{2233}^{p}\right)}^{2}}=C_{1111}^{p}-\frac{2{\left(C_{1122}^{p}\right)}^{2}}{C_{2222}^{p}+C_{2233}^{p}},\\ E_{2}^{p}=E_{3}^{p}=\frac{\Delta }{C_{1111}^{p}C_{2222}^{p}-{\left(C_{1122}^{p}\right)}^{2}},\\ \nu_{12}^{p}=\nu_{13}^{p}=\frac{C_{1122}^{p}\left(C_{2222}^{p}-C_{2233}^{p}\right)}{C_{1111}^{p}C_{2233}^{p}-{\left(C_{1122}^{p}\right)}^{2}},\;\nu_{31}^{p}=\nu_{21}^{p}=\frac{C_{1122}^{p}}{C_{2222}^{p}+C_{2233}^{p}},\\ \nu_{23}^{p}=\nu_{32}^{p}=\frac{C_{1111}^{p}C_{2233}^{p}-{\left(C_{1122}^{p}\right)}^{2}}{C_{1111}^{p}C_{2222}^{p}-{\left(C_{1122}^{p}\right)}^{2}},\\ G_{12}^{p}=G_{31}^{p}=C_{1212}^{p}=C_{3131}^{p},\;G_{23}^{p}=C_{2323}^{p}=\frac{1}{2}\left(C_{2222}^{p}-C_{2233}^{p}\right),\\ \Delta =\left(C_{2222}^{p}-C_{2233}^{p}\right)\left[C_{1111}^{p}\left(C_{2222}^{p}+C_{2233}^{p}\right)-2{\left(C_{1122}^{p}\right)}^{2}\right]. \end{array}$$

When the liquid-filled polymer composition has hardened, the porous transversely isotropic material is linearly elastic at small strains; therefore, its load-carrying element is also assumed linearly elastic in this strain range and its stress–strain relation is described by the straight line $\overset{*}{\sigma }={\overset{*}{E}}_{1}\overset{*}{\varepsilon }$, where ${\overset{*}{E}}_{1}$ is the elastic modulus.

## 3. Nonlinearity

If the load-carrying element is nonlinearly elastic outside the strain interval $\left[\bar{\varepsilon },\;\overset{+}{\varepsilon }\right]$, its nonlinear deformation is described by an appropriate nonlinear function. For this aim, e.g., parabolic and ellipsoidal functions can be used. 

### 3.1. Parabolic Functions

If $\overset{*}{\varepsilon }>\overset{+}{\varepsilon }$, then

(15) $$\overset{*}{\sigma }=\overset{+}{\sigma_{0}}+\left(\overset{+}{\sigma }-\overset{+}{\sigma_{0}}\right){\left[{\left(\frac{\overset{*}{\varepsilon }-\overset{+}{\varepsilon_{0}}}{\overset{+}{\varepsilon }-\overset{+}{\varepsilon_{0}}}\right)}^{2}\right]}^{\frac{\overset{+}{n}}{2}},$$

if $\overset{*}{\varepsilon }<\bar{\varepsilon }$, then

(16) $$\overset{*}{\sigma }=\bar{\sigma_{0}}+\left(\bar{\sigma }-\bar{\sigma_{0}}\right){\left[{\left(\frac{\overset{*}{\varepsilon +}\bar{\varepsilon_{0}}}{\bar{\varepsilon }+\bar{\varepsilon_{0}}}\right)}^{2}\right]}^{\frac{\bar{n}}{2}},$$

$$\left\{\begin{array}{ll} 0\leq \overset{+}{\varepsilon_{0}}<\overset{+}{\varepsilon }, & \;\bar{\varepsilon }<\bar{\varepsilon_{0}}\leq 0,\\ 0\leq \overset{+}{\sigma_{0}}<\overset{+}{\sigma }, & \;\bar{\sigma }<\bar{\sigma_{0}}\leq 0. \end{array}\right.$$

The points $\left(\overset{\pm }{\varepsilon_{0}},\;\overset{\pm }{\sigma_{0}}\right)$ are vertices of the parabolas. The parabolas join the straight line at the points $\left(\overset{\pm }{\varepsilon },\;\overset{\pm }{\sigma }\right)$.

If it is required that the straight line be tangential to the parabolas at the points $\left(\overset{\pm }{\sigma },\;\overset{\pm }{\varepsilon }\right)$, then the powers $\overset{\pm }{n}$ have to satisfy the relation

(17) $$\overset{\pm }{n}=\frac{\overset{\pm }{\varepsilon }-\overset{\pm }{\varepsilon_{0}}}{\overset{\pm }{\sigma }-\overset{\pm }{\sigma_{0}}}{\overset{*}{E}}_{1}.$$

**Figure 2:**
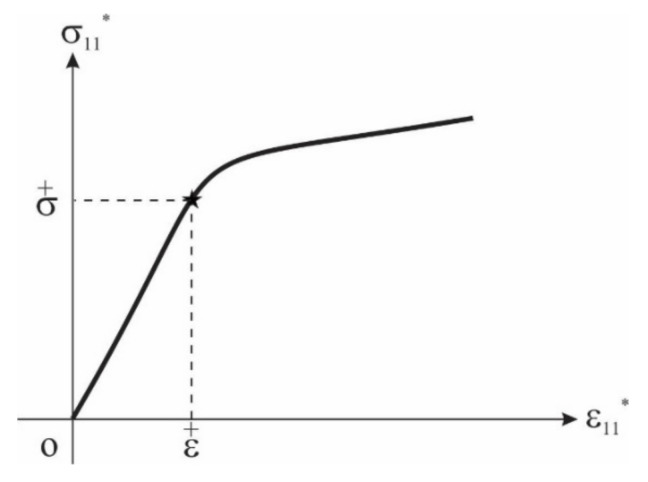
Nonlinear stress–strain relation of the load-carrying element.

This case is illustrated in Figure 2.

If $\overset{\pm }{\varepsilon_{0}}=0$ and ${\overset{\pm }{\sigma }}_{0}=0$, then $\overset{\pm }{n\;}=1$, and the parabolas degenerate into the straight line $\overset{*}{\sigma }={\overset{*}{E}}_{1}\overset{*}{\varepsilon }$. In general, the parameters $\overset{\pm }{\varepsilon_{0}}$ and $\overset{\pm }{\sigma_{0}}$ depend on the volume fraction $V_{f}$ of clay nanoparticles in the polymer. Then, they can be expressed as

(18) $$\overset{\pm }{\varepsilon_{0}}={\overset{\pm }{\varepsilon }}_{00}\exp\left(\overset{\pm }{n_{\varepsilon }}V_{f}\right),\;\overset{\pm }{\sigma_{0}}={\overset{\pm }{\sigma }}_{00}\exp\left(\overset{\pm }{n_{\sigma }}V_{f}\right).$$

### 3.2. Elliptic Functions

If $\left|\overset{+}{\varepsilon }\right|>\left|\overset{\pm }{\varepsilon }\right|$, then

(19) $$\overset{\pm }{\sigma }=\overset{\pm }{b_{c}}\pm \frac{\overset{\pm }{b}}{\overset{\pm }{a}}\sqrt{{\overset{\pm }{a}}^{2}-{\left(\overset{*}{\varepsilon }-{\overset{\pm }{a}}_{c}\right)}^{2}}$$

are ellipses with semiaxes $\overset{\pm }{a}$ and $\overset{\pm }{b}$ and centers $\left({\overset{\pm }{a}}_{c},\;{\overset{\pm }{b}}_{c}\right)$. 

They pass through the points $\left(\overset{\pm }{\varepsilon },\;\overset{\pm }{\sigma }\right)$ if

(20) $$\overset{\pm }{b_{c}}=\overset{\pm }{\sigma }\mp \frac{\overset{\pm }{b}}{\overset{\pm }{a}}\sqrt{{\overset{\pm }{a}}^{2}-{\left(\overset{\pm }{\varepsilon }-{\overset{\pm }{a}}_{c}\right)}^{2}}.$$

If it is required that the straight line $\overset{*}{\sigma }={\overset{*}{E}}_{1}\overset{\pm }{\varepsilon }$ be the tangent to the ellipses at the points $\left(\overset{\pm }{\varepsilon },\;\overset{\pm }{\sigma }\right)$, then the parameters $\overset{\pm }{b}$ have to obey the relation

(21) $$\overset{\pm }{b}=\overset{\pm }{\sigma }\mp \frac{\overset{\pm }{a}\sqrt{{\overset{\pm }{a}}^{2}-{\left(\overset{\pm }{\varepsilon }-{\overset{\pm }{a}}_{c}\right)}^{2}}}{\overset{\pm }{\varepsilon }-{\overset{\pm }{a}}_{c}}{\overset{*}{E}}_{1}.$$

If $V_{f}>0$, then $\overset{\pm }{a}$, ${\overset{\pm }{a}}_{c}$, and ${\overset{*}{E}}_{1}$ are functions of $V_{f}$, e.g.,

(22) $$\overset{\pm }{a}={\overset{\pm }{a}}_{0}\exp\left(\overset{\pm }{k_{a}}V_{f}\right),\;{\overset{\pm }{a}}_{c}={\overset{\pm }{a}}_{c\left(0\right)}\exp\left(\overset{\pm }{k_{c}}V_{f}\right).$$

If the data for nonlinear deformation of a structural element with $V_{f}>0$ are not available, then the function $\overset{*}{\sigma }(\overset{*}{\varepsilon })$ can be approximately predicted employing the parabolic or elliptic function indicated, assuming that the parameters $\overset{\pm }{\varepsilon }$ do not depend on $V_{f}$ and ${\overset{\pm }{n}}_{\varepsilon }={\overset{\pm }{n}}_{\sigma }=0$ in relations (18) or ${\overset{\pm }{k}}_{a}={\overset{\pm }{k}}_{b}=0$ in relations (22) and considering the fact that the value of $V_{f}$ is typically rather small. Then, $V_{f}$ will affect mainly the elastic modulus ${\overset{*}{E}}_{1}$.

## 4. Comparison of Theoretical Calculations with Experimental Data

As an example of nonlinear deformation of a transversely isotropic cellular plastic, we calculated its tension diagram along the $x_{2}$ axis at $\varepsilon_{22}>0$, $\varepsilon_{11}=-\nu_{12}\varepsilon_{22}$, $\varepsilon_{33}=-\nu_{32}\varepsilon_{22}$, and $\sigma_{11}=\sigma_{33}=0$. Then,

(23) $$\begin{array}{c} \overset{*}{\varepsilon_{11}}=\left(-\nu_{12}l_{11}^{2}+l_{12}^{2}-\nu_{32}l_{13}^{2}\right)\varepsilon_{22},\\ \overset{*}{\varepsilon_{13}}=\left(l_{12}l_{22}-\nu_{32}l_{23}\right)\varepsilon_{22},\\ \overset{*}{\varepsilon_{12}}=0. \end{array}$$

Young’s modulus $E_{m}$ = 2300 MPa and the nonlinear deformation parameters, Equation (19), of the monolithic polymer were obtained from experimental stress–strain diagrams of the neat PUR polymer in tension [29,30], while the Poisson’s ratio was taken as $\nu_{m}$ = 0.32 [31]. The volume fraction $V_{f}$ of the filler was evaluated from the weight fraction *W_f_* as $V_{f}=W_{f}\rho_{m}/\left[W_{f}\rho_{m}+\left(1-W_{f}\right)\rho_{f}\right]$, using the densities of monolithic PUR $\rho_{m}$ reported in [29] and montmorillonite clay $\rho_{f}$ [32]. For filled foams, it was assumed that Cloisite^®^ 30B clay filler had fully exfoliated during the rigid PUR foam production; geometrical dimensions and mechanical characteristics of the clay nanoplatelets used in calculation were taken from [31]. Introduction of clay filler led to a slight reduction of cell size, while the geometrical anisotropy of the foam cells remained the same within the experimental scatter [33] (see also SEM images of neat [30] and filled [33] foams), therefore the average value of cell shape anisotropy ratio (i.e., the degree of cell extension) *k* = 1.5 [30,33] was used in modeling.

Predicted deformation diagrams are compared with the experimental data, reported in [34], of neat and Cloisite^®^ 30B clay-filled foams with the same composition of the PUR matrix as the monolithic polymer [30]. The formulation and manufacturing procedure of foams, as well as preparation and testing of foam specimens, are described in [34]. For completeness and ease of reference, we briefly recapitulate the relevant information. Foam specimens of dog-bone shape, with a rectangular test section of 85 mm length, 22 mm width and 20 mm thickness, where cut from slices of the free-rise foam blocks so that the mechanical response in the direction normal to foam rise could be characterized. Metallic plates with hooks were glued to the ends of the specimens, and short chains were attached to the hooks to enable gripping. Such a gripping system [29] provided alignment of the specimen with the line of action of the applied load, eliminating bending and ensuring pure tension during the test. A clip-on extensometer with 50 mm base length was used for strain measurement in the loading direction. Tensile tests were performed at a displacement rate of 8 mm/min.

**Figure 3:**
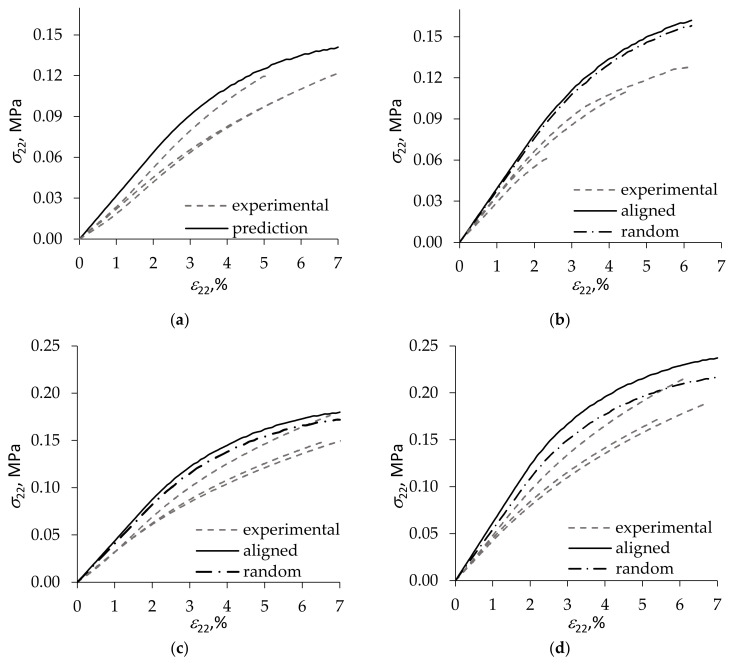
Stress–strain diagrams in tension transverse to the rise direction of ca. 40 kg/m^3^ density foams with (**a**) 0, (**b**) 1, (**c**) 2, and (**d**) 5 wt.% loading of Cloisite^®^ 30B clay. Foam test results are plotted by dashed lines, and model predictions—by solid lines for clay platelets aligned with strut axis and by dash-dot lines for a random orientation of clay platelets in foam struts.

The test results in tension transverse to the foam rise direction for foams of ~40 kg/m^3^ density, with 0, 1, 2, and 5 wt.% loading of the clay filler [34], are shown in Figure 3 together with the model prediction obtained using the elliptic nonlinear function (19) with ${\overset{\pm }{k}}_{a}={\overset{\pm }{k}}_{b}=0$. Three foam specimens were tested for each filler loading level; the respective experimental stress–strain diagrams are plotted by dashed lines in Figure 3.

Two cases of the spatial orientation of clay nanoplatelets are considered in modeling—random orientation and perfect alignment of the platelets along strut axis. The alignment occurs during foaming, due to suction of the liquid chemical formulation and filler particles through the Gibbs’ channels between the growing bubbles and stretching of the filled polymeric struts along their longitudinal axis [35,36]. 

**Figure 4:**
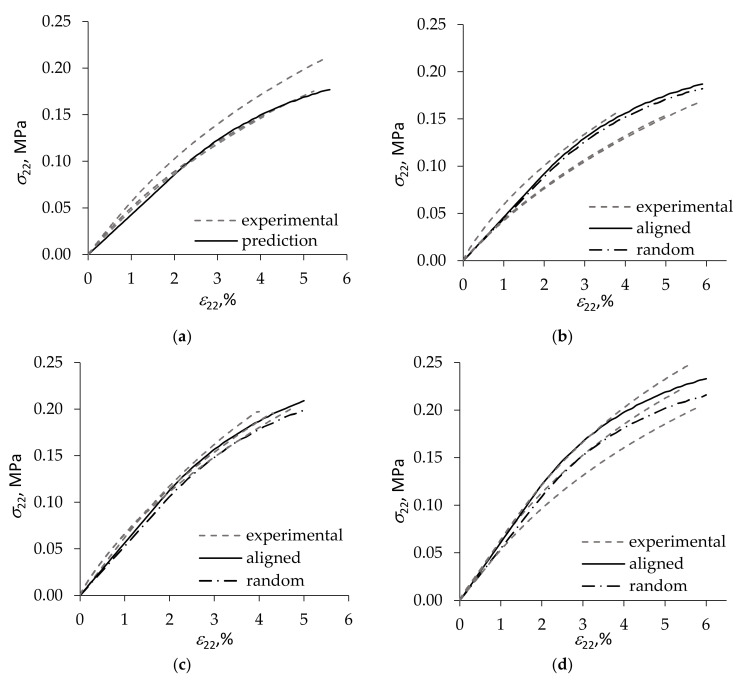
Stress–strain diagrams in tension transverse to the rise direction of ca. 50 kg/m^3^ density foams with (**a**) 0, (**b**) 1, (**c**) 2, and (**d**) 3 wt.% loading of Cloisite^®^ 30B clay. Foam test results are plotted by dashed lines, and model predictions—by solid lines for clay platelets aligned with strut axis and by dash-dot lines for a random orientation of clay platelets in foam struts.

Numerical calculations showed that, as expected, alignment of the anisometric filler in the axial direction of foam struts increased foam stiffness compared with the case of random orientation, see Figure 3b–d. The effect of alignment became more pronounced with increasing clay loading. The same qualitative tends are seen also in Figure 4 presenting comparison of test results of rigid PUR foams with a slightly greater, ~50 kg/m^3^, density and 0, 1, 2, and 3 wt.% loading of Cloisite^®^ 30B [34] with theoretical prediction performed as described above.

TEM microscopy has revealed that clay nanoplatelet-filled cellular plastics, e.g., polypropylene foams [6], represent an intermediate case, when a certain amount of the anisometric nanoparticles remains randomly oriented in the central part of load-carrying elements, especially knots. The stress–strain diagrams of such materials are expected to lie between the diagrams of materials comprising irregularly oriented and perfectly aligned nanoplatelets.

It is seen in Figure 3b–d that the calculated $\sigma_{22}\left(\varepsilon_{22}\right)$ curves for filled foams tend to pass above the experimental diagrams. Such an overestimation of foam stiffness by the model is likely to be caused by a partial exfoliation of the clay contained in foam struts, as opposed to complete exfoliation assumed in calculations. It has been shown in, e.g., [37] that, for a given loading of clay filler, reduction in the fraction of exfoliated particles leads to a substantial decrease of the stiffness of the filled composite. In addition, the nonlinearity of deformation appears overestimated at the higher loadings of the filler, Figure 3c,d and Figure 4d. This can be explained by the fact that, when describing nonlinear deformation of foams, the effect of $V_{f}$ was taken into account only approximately due to the simplifying assumption used that ${\overset{\pm }{k}}_{a}={\overset{\pm }{k}}_{b}=0$. To fully reflect in the model the effect of filler on the nonlinearity of foam deformation, it is necessary to determine the coefficients ${\overset{\pm }{k}}_{a}$ and ${\overset{\pm }{k}}_{b}$ in relations (22) for some non-zero value of $V_{f}$, thus calibrating the model.

## 5. Conclusions

A structural model for a nonlinearly deforming highly porous cellular plastic filled with clay nanoplatelets is derived taking into account the spatial alignment of the anisometric particles with respect to the strut axis attained during foaming. The model relates nonlinearity in foam deformation to nonlinearly elastic response of foam strut material. Reasonable agreement of model prediction with experimental data is demonstrated for tensile deformation of neat and clay nanoplatelet-filled highly porous rigid polyurethane foams. For neat foams, this suggests that the axial stress-strain response of a foam strut is close to that of a monolithic polymer specimen of a macroscopic size. For composite foams, alignment of the clay platelets with the axial direction of foam struts increased the predicted foam stiffness compared with that for random orientation of platelets. This effect became more pronounced at greater clay loadings. The theoretical deformation diagrams of composite foams where close to or above the experimental ones. As complete exfoliation of the clay particles was assumed in the model, the predicted stiffness of foams being higher than the experimental is likely to stem from only partial exfoliation of clay achieved during manufacture of filled foams.

## Data Availability

The data presented in this study are available on request from the corresponding author.

## References

[1] Shen J., Han X., Lee L.J. (2006). Nanoscaled reinforcement of polystyrene foams using carbon nanofibers. J. Cell. Plast..

[2] Hussain S., Kortschot M. (2014). Polyurethane foam mechanical reinforcement by low-aspect ratio microcrystalline cellulose and glass fibres. J. Cell. Plast..

[3] Shaayegan V., Ameli A., Wang S., Park C.B. (2016). Experimental observation and modeling of fiber rotation and translation during foam injection molding of polymer composites. Compos. Part A.

[4] Wang S., Ameli A., Shaayegan V., Kazemi Y., Huang Y., Naguib H.E., Park C.B. (2018). Modelling of rod-like fillers’ rotation and translation near two growing cells in conductive polymer composite foam processing. Polymers.

[5] Andersons J., Kirpluks M., Cabulis U. (2020). Reinforcement efficiency of cellulose microfibers for the tensile stiffness and strength of rigid low-density polyurethane foams. Materials.

[6] Wang L., Yang B., Zhou L., Xue B., Yang Z. (2021). Evolution of anisotropic bubbles and transition of the mechanical and electrical properties during a non-continuous two-step foaming of epoxy/carbon nanofiber composites. Compos. Sci. Technol..

[7] Okamoto M., Nam P.H., Maiti P., Kotaka T., Nakayama T., Takada M., Ohshima M., Usuki A., Hasegawa N., Okamoto H. (2001). Biaxial flow-induced alignment of silicate layers in polypropylene/clay nanocomposite foam. Nano Lett..

[8] Nam P.H., Maiti P., Okamoto M., Kotaka T., Nakayama T., Takada M., Ohshima M., Usuki A., Hasegawa N., Okamoto H. (2002). Foam processing and cellular structure of polypropylene/clay nanocomposites. Polym. Eng. Sci..

[9] Shishkina O., Lomov S.V., Verpoest I., Gorbatikh L. (2015). Modeling of elastic properties of cell-wall material in nanoclay-reinforced foams. J. Cell. Plast..

[10] Walter T.R., Richards A.W., Subhash G. (2009). A unified phenomenological model for tensile and compressive response of polymeric foams. J. Eng. Mater. Technol..

[11] Ahern A., Verbist G., Weaire D., Phelan R., Fleurent H. (2005). The conductivity of foams: A generalisation of the electrical to the thermal case. Colloids Surf. A Physicochem. Eng. Asp..

[12] Huber A.T., Gibson L.J. (1988). Anisotropy of foams. J. Mater. Sci..

[13] Gibson L.J., Ashby M.F. (1997). Cellular Solids: Structure and Properties.

[14] Dement’ev A.G., Tarakanov O.G. (1970). Model analysis of the cellular structure of plastic foams of the polyurethane type. Polym. Mech..

[15] Gong L., Kyriakides S., Jang W.-Y. (2005). Compressive response of open-cell foams. Part I: Morphology and elastic properties. Int. J. Solids Struct..

[16] Sullivan R.M., Ghosn L.J., Lerch B.A. (2008). A general tetrakaidecahedron model for open-celled foams. Int. J. Solids Struct..

[17] Sullivan R.M., Ghosn L.J. (2009). Shear moduli for non-isotropic, open cell foams using a general elongated Kelvin foam model. Int. J. Eng. Sci..

[18] Sullivan R.M., Ghosn L.J., Lerch B.A. (2009). Application of an elongated Kelvin model to space shuttle foams. J. Spacecr. Rocket..

[19] Kontou E., Spathis G., Kefalas V. (2012). Statistical model for the compressive response of anisotropic polymeric and metallic foams. J. Mater. Sci..

[20] Lagzdins A., Zilaucs A., Beverte I., Andersons J. (2013). Calculating the elastic constants of a highly porous cellular plastic with an oriented structure. Mech. Compos. Mater..

[21] Gong L., Kyriakides S. (2005). Compressive response of open-cell foams. Part II: Initiation and evolution of crushing. Int. J. Solids Struct..

[22] Gaitanaros S., Kyriakides S., Kraynik A.M. (2012). On the crushing response of random open-cell foams. Int. J. Solids Struct..

[23] Iizuka M., Goto R., Siegkas P., Simpson B., Mansfield N. (2021). Large deformation finite element analyses for 3D X-ray CT scanned microscopic structures of polyurethane foams. Materials.

[24] Spathis G., Kontou E. (2011). Modeling the compressive stress–strain response of polymeric foams. J. Appl. Polym. Sci..

[25] Lagzdins A., Zilaucs A., Beverte I., Andersons J. (2012). A refined strut model for calculating the elastic constants of highly porous cellular plastics by the method of orientational averaging. Mech. Compos. Mater..

[26] Lagzdins A., Zilaucs A., Beverte I., Andersons J. (2016). Estimation of the elastic constants of highly porous cellular plastics reinforced with fibres embedded in foam struts. J. Compos. Mater..

[27] Lagzdins A., Maksimov R.D., Plume E. (2006). Elasticity of composites with irregularly oriented shape-anisotropic filler particles. Mech. Compos. Mater..

[28] Maksimov R.D., Gaidukovs S., Kalnins M., Zicans J., Plume E. (2006). A nanocomposite based on a styrene-acrylate co-polymer and native montmorillonite clay. 2. Modeling the elastic properties. Mech. Compos. Mater..

[29] Cabulis U., Sevastyanova I., Andersons J., Beverte I. (2014). Rapeseed oil-based rigid polyisocyanurate foams modified with nanoparticles of various type. Polimery.

[30] Andersons J., Kirpluks M., Stiebra L., Cabulis U. (2016). Anisotropy of the stiffness and strength of rigid low-density closed-cell polyisocyanurate foams. Mater. Des..

[31] Maksimov R.D., Plume E. (2012). Elastic properties of a polyurethane/montmorillonite nanocomposite. Mech. Compos. Mater..

[32] Osipov V.I. (2012). Density of clay minerals. Soil Mech. Found. Eng..

[33] Andersons J., Cābulis U., Stiebra L., Kirpļuks M., Spārniņš E. (2017). Modeling the mode I fracture toughness of anisotropic low-density rigid PUR and PIR foams. Int. J. Fract..

[34] Cābulis U., Kirpļuks M., Andersons J. (2013). The effect of montmorillonite type nanoparticles on stiffness and flammability of rapeseed oil based polyisocyanurate foams. Key Eng. Mater..

[35] Klempner D., Frisch K.C. (1991). Handbook of Polymeric Foams and Foam Technology.

[36] Berlin A.A., Shutov F.A. (1980). Chemistry and Technology of Gas-Filled High-Polymers.

[37] Zicāns J., Maksimov R.D., Plūme E., Merijs Meri R., Jansons J. (2018). The effect of partial exfoliation of multilayer silicate filler particles on the elastic properties of a polymer composite. Compos. Struct..

